# 522. Evaluation of Three COVID-19 Monoclonal Antibody Regimens in the Context of Rising B.1.526 Prevalence in New York City

**DOI:** 10.1093/ofid/ofab466.721

**Published:** 2021-12-04

**Authors:** Hongkai Bao, Yi Guo, Kelsie Cowman, Victor Chen, Priya Nori, Priya Nori

**Affiliations:** 1 Montefiore Medical Center, The Bronx, New York; 2 Montefiore Medical Center, Albert Einstein College of Medicine, Bronx, NY; 3 Montefiore Medical Center and Albert Einstein College of Medicine, New York, NY; 4 UC San Diego Health, San Diego, CA; 5 Montefiore Medical Center/Albert Einstein College of Medicine, Bronx, NY

## Abstract

**Background:**

Monoclonal antibodies were given emergency use authorization (EUA) by the Food and Drug Administration for the treatment of high-risk, outpatient COVID-19 infection. In New York City (NYC), the emergence and rapid growth of the B.1.526 variant of concern (VOC) possessing the E484K mutation was first noted in February 2021. *In-vitro* studies subsequently confirmed attenuated monoclonal antibody neutralization against VOCs. At our institution, bamlanivimab (BAM) alone or with etesevimab (B/E) and casirivimab/imdevimab (C/I) were utilized at different phases of the pandemic. The objective of this study was to assess their comparative efficacies in a highly variant prevalent setting.

**Methods:**

This retrospective analysis was conducted at an urban hospital in the Bronx, NY and evaluated adult monoclonal antibody recipients from any of our infusion sites. Patients initially received BAM but given the high prevalence of variants, treatment was transitioned to first B/E and then C/I exclusively. We compared BAM versus combination therapy as well as B/E versus C/I individually. The primary outcome was all-cause hospital admission within 30 days post infusion.

**Results:**

From February 1 to March 7, 2021, 358 patients received BAM and from March 17 to May 9, 2021, 86 and 179 patients received B/E and C/I, respectively. Compared to any combination infusion, patients who received BAM were significantly older, more likely to possess ≥ 2 qualifying EUA criteria, and less likely to be vaccinated for COVID-19 prior to infusion (Table 1). Following B/E and C/I, 4.5% of patients were admitted versus 10.1% for BAM, *p*=0.011. There were no significant differences in admission between B/E and C/I recipients, *p*=0.485. After excluding fully vaccinated patients (n=14) and adjusting for age and ≥ 2 EUA criteria, combination therapy remained associated with decreased odds of hospitalization compared to BAM (odds ratio, 0.48; 95% confidence interval, 0.24-0.94).

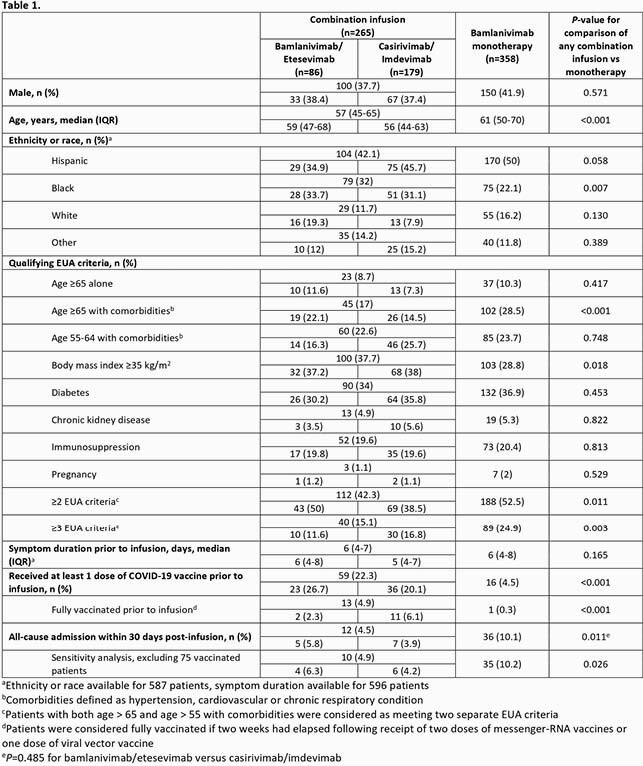

**Conclusion:**

Combination therapy may be associated with fewer hospital admissions following infusion, although there were no statistically significant differences between the individual combination infusions. We suggest similar studies be conducted by other sites to understand the clinical impact of local SARS-CoV-2 variants on antibody efficacy.

**Disclosures:**

**Yi Guo, PharmD, BCIDP**, Merck (Research Grant or Support) **Kelsie Cowman, MPH**, Merck (Research Grant or Support) **Priya Nori, MD**, Merck (Grant/Research Support) **Priya Nori, MD**, Nothing to disclose

